# First-Principles Study of Titanium-Doped B_7_ Cluster for High Capacity Hydrogen Storage

**DOI:** 10.3390/molecules29235795

**Published:** 2024-12-07

**Authors:** Haishen Huang, Guoxu Li, Zhenqiang Li, Tingyan Zhou, Ping Li, Xiude Yang, Bo Wu

**Affiliations:** 1 School of Physics and Electronic Science, Zunyi Normal University, Zunyi 563006, China; haishenh@yeah.net (H.H.);; 2 ZNB Quality of Scientific Research Academy, Haikou 571152, China

**Keywords:** boron cluster, DFT, titanium doping, transition state, hydrogen storage

## Abstract

The geometrical structure, stability, electronic properties, and hydrogen storage capabilities of a titanium-doped B_7_ cluster was calculated using density functional theory computations. The results show that the TiB_7_ cluster is predicted to be stable under near-ambient conditions based on an ab initio molecular dynamic simulation. The transition state analysis found that the H_2_ molecule can dissociate on the TIB_7_ cluster surface to form a hydride cluster. The Ti atom within the TiB_7_ cluster demonstrates an impressive capacity to adsorb up to five H_2_ molecules, achieving a peak hydrogen storage mass fraction of 7.5%. It is worth noting that the average adsorption energy of H_2_ molecules is 0.27–0.32 eV, which shows that these configurations are suited for reversible hydrogen storage under mild temperature and pressure regimes. In addition, calculations found that both polarization and hybridization mechanisms play pivotal roles in facilitating the adsorption of H_2_ molecules onto the TiB_7_ cluster. Our research results show that the TiB_7_ cluster has potential for hydrogen storage applications under near-ambient conditions.

## 1. Introduction

In light of the rapid depletion of traditional fossil fuels and their severe global environmental implications, the exploration of innovative materials for sustainable energy has garnered significant theoretical and experimental interest over the past two decades [1,2,3]. Hydrogen energy, being the cleanest, most sustainable, and renewable form of energy, is anticipated to gradually diminish our reliance on fossil fuels [4,5]. Nonetheless, the absence of secure and efficient storage solutions poses a significant challenge to the practical utilization of hydrogen [6]. To address this issue, it is imperative to develop hydrogen storage materials with high gravimetric and volumetric densities that allow for the reversible storage and release of hydrogen under moderate temperature and pressure conditions. An optimal hydrogen storage system would maintain hydrogen in molecular form, with an average H_2_ molecular binding energy within the range of 0.2–0.6 eV [7]. Therefore, seeking and engineering novel materials are crucial for hydrogen energy applications.

Owing to its electron-deficient bonding, boron is an element of unique structural complexity. Similar to carbon, boron can form a variety of nanostructures: clusters, fullerenes, nanotubes, and nanosheets [8,9]. For *n* = 2 to 14, boron clusters favor quasi-planar or convex structures, which can be regarded as fragments of fullerenes or nanotubes or nanosheets predicted by Boustani in 1997 [10]. For larger sizes, Tai et al. reported that both B_26_ and B_27_ clusters adopt tubular configurations, whereas B_28_ and B_29_ manifest as quasi-planar geometries [11]. Some special sizes such as B_40_ exhibit a fullerene form [12]. Recently, the formation of two types of two-dimensional boron sheets on Ag (111) was confirmed by experiments and first-principles calculations [13]. All these nanostructures have a high ratio of surface to volume, which may enhance the capacity of hydrogen storage and release.

Over the past two decades, metal-doped boron nanomaterials have garnered significant theoretical and experimental interest due to their unique physical and chemical characteristics [14,15], as well as their hydrogen storage capabilities [16,17,18,19]. For example, Du et al. presented a theoretical exploration of the Saturn-like charge-transfer complex Li_4_&B_36_, wherein each lithium atom exhibits the capacity to associate with as many as six hydrogen molecules, albeit through relatively weak binding interactions characterized by average energies ranging from 0.08 eV to 0.14 eV. This complex boasts an impressive gravimetric hydrogen storage density, reaching up to 10.4% [20]. Chen et al. conducted a study using density functional theory (DFT) to investigate the reversible hydrogen storage potential of both pristine and lithium-decorated boron hydride sheets. Their findings revealed that the addition of lithium to the boron hydride sheet significantly enhanced its hydrogen storage capacity, achieving a mass fraction of 11.57%. This enhancement markedly surpassed the storage capability of the undecorated sheet [21].

Recently, transition-metal-decorated nanomaterials, which serve as promising hydrogen storage media, have become a research hotspot [22]. For instance, based on DFT calculations, *M*-decorated (*M* = Sc, Ti, Fe, Co, Ni) B_38_ fullerenes could store more than 7.2 wt% of hydrogen molecules [23,24,25]. Our group previously reported the structural, electronic, and spectral properties of ScB_*n*_ (*n* = 1–12) clusters. Notably, we observed a pronounced red-shift in the absorption spectrum as the number of adsorbed hydrogen molecules escalated, which provides a "fingerprint" of hydrogen storage capacity [26]. As an important industrial material, elemental Ti has attracted special theoretical and experimental attention motivated by its excellent physical and chemical properties including good corrosion resistance, ductility, and high thermal conductivity. Morozova et al. reported experimental evidence for a Ti/B nanocomposite as a hydrogen storage material [27]. Furthermore, titanium is often used as a doping element to enhance the hydrogen storage kinetics of nanomaterials [28]. For example, Li et al. discovered that a single titanium atom is capable of associating with up to four hydrogen molecules. They emphasized that the Kubas interaction alongside charge transfer between the hydrogen molecule and titanium atom are pivotal in determining the optimal binding energies [29]. In 2011, we studied the ground-state configurations of Ti-doped B_*n*_ (*n* = 1–12) clusters, leading us to identify the stability magic numbers as 3, 7, and 10 [30]. However, despite these advancements, there is still a limited understanding of the hydrogen storage capabilities exhibited by these titanium-doped small boron clusters.

In this study, a thorough theoretical exploration of the Ti-doped B_7_ cluster’s geometry, stability, electronic properties, and hydrogen storage capabilities was conducted using density functional theory calculations. Our findings reveal that the TiB_7_ cluster possesses sufficient stability to adsorb up to five H_2_ molecules, achieving a remarkable hydrogen storage mass fraction of 7.5%. The average adsorption energy of these H_2_ molecules falls in the desirable range of 0.27–0.32 eV, indicating that these configurations are well suited for reversible hydrogen storage under moderate temperature and pressure conditions.

## 2. Results and Discussion

### 2.1. Geometry and Stability of TiB_7_ Cluster

**Figure 1:**
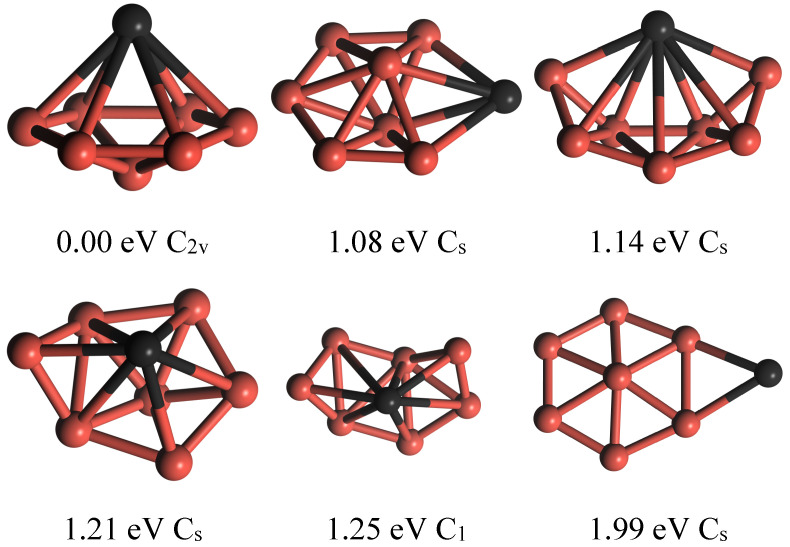
The geometry structures of the stable TiB_7_ clusters.

The initial structures of Ti-doped boron clusters can either draw inspiration from the structural models of metal-doped boron clusters reported in the existing literature [1,14,22] or be achieved by capping, filling, or replacing boron atoms in pure boron clusters with Ti atoms. By structural optimization, in the absence of symmetry limitations, the stable structures of the Ti-doped B_7_ cluster can be obtained. The lowest-energy (ground-state) structure, characterized by the $C_{2v}$ symmetry depicted in Figure 1, aligns closely with our prior GGA PW91 computations [30] and Wang et al.’s BPBE calculations [31]. The stable structures’ relative energies exhibit some differences between the current study and previous works, highlighting the significance of electronic correlations in boron clusters. The results indicate that all TiB_7_ structures, including the following TiB_7_(H)_6_ and TiB_7_(H)_7_ clusters, are devoid of imaginary frequencies, and those with lower spin multiplicity possess lower total energies. Specifically, the ground-state structures of TiB_7_ and TiB_7_(H)_6_ clusters exhibit a doublet spin configuration, whereas the ground-state TiB_7_(H)_7_ clusters show a singlet spin configuration. This consistency arises when hydrogen adsorption occurs, as the even number of electrons in hydrogen molecules does not alter the parity of the total electron number in the clusters, thereby preserving the spin configuration of their ground states. All structures and frequency data are provided in the Supplementary Materials.

**Figure 2:**
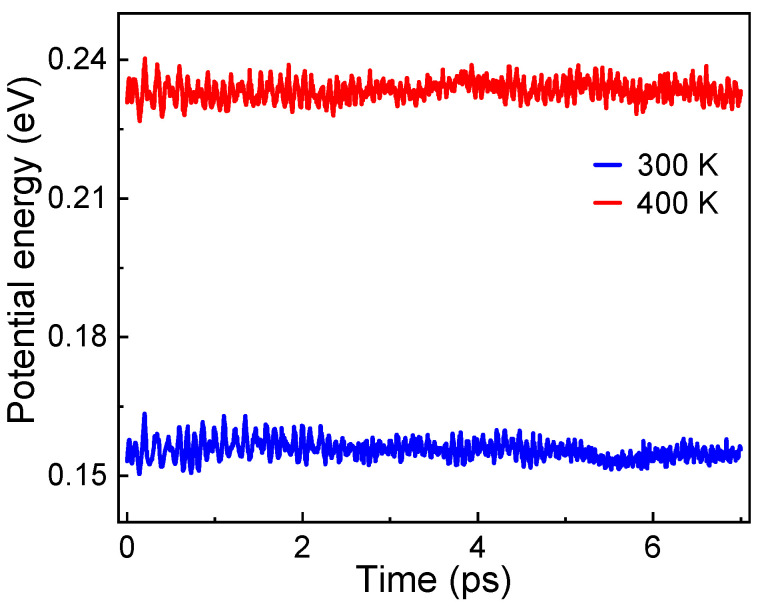
Potential energy trajectories at 300 K and 400 K for TiB_7_ cluster. Energy values are calculated in relation to ground-state structure, which represents state at absolute zero (0 K).

The PW91 and BPBE calculations of the previous works both pointed out that the ground-state TiB_7_ cluster is the magic number cluster due to its extra stability [30,31]. To further confirm this remarkable stability, in the present work, an ab initio molecular dynamic (AIMD) simulation was performed in a constant volume and temperature (*NVT*) ensemble, using a time step of 1 fs for the integration. The thermodynamic stability of the ground-state TiB_7_ cluster above room temperature (300 K and 400 K) was studied with a time-scale of 7 ps. The temporal variation of potential energies is illustrated in Figure 2. After 7 ps of AIMD simulation, the fluctuations in energy at both 300 K and 400 K are negligible, and the ground-state structure of TiB_7_ remains unchanged, indicating the cluster is relatively stable under near-ambient conditions and can keep its original configuration during H_2_ molecule adsorption and dissociation.

### 2.2. Adsorption and Dissociation of H_2_ Molecule on TiB_7_ Cluster

#### 2.2.1. Dissociation of H_2_ Molecule on TiB_7_ Cluster

**Figure 3:**
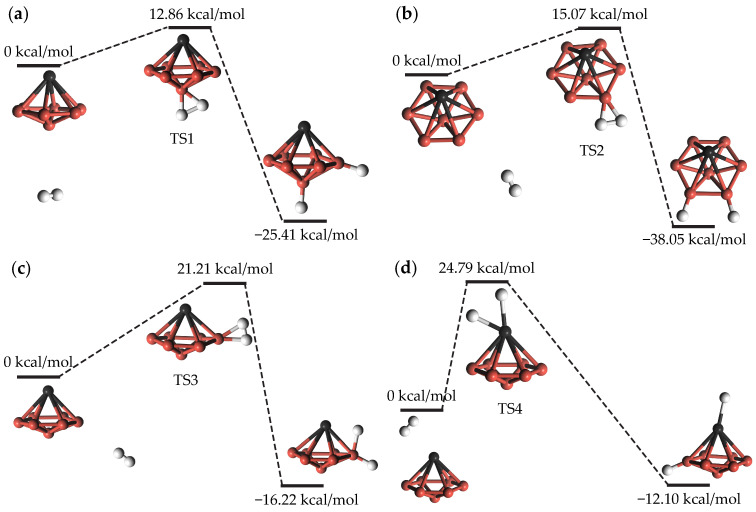
The reactant, transition state, and product structures of the four reaction paths for hydrogen molecular dissociation on the TiB_7_ cluster. (**a**) Pathway 1, (**b**) Pathway 2, (**c**) Pathway 3, and (**d**) Pathway 4. The free energy values are relative to reactants.

Our initial investigation focused on the dissociation mechanism of the H_2_ molecule on the TiB_7_ cluster, a crucial aspect for understanding hydrogen storage capabilities [32]. Within the TiB_7_ cluster, there are two types of B atoms: those located on the edge of the boron hexagon (B1) and the one situated in the center of the hexagon (B2). We considered various orientations and positions of the H_2_ molecule as it approached the TiB_7_ surface. These orientations can be broadly categorized into three main approaches: toward the B2 atom, the B1 atom, and the Ti atom. For each approach, we constructed initial reactant structures by placing the H_2_ molecule in close proximity to the TiB_7_ surface. Similarly, for the products, we designed the initial structures based on the binding situations of the two dissociated H atoms with the Ti, B1, and B2 atoms. The four possible pathways of H_2_ molecule dissociation on the TiB_7_ cluster, designated as Pathway 1, Pathway 2, Pathway 3, and Pathway 4, respectively, were obtained and are shown in Figure 3. The reaction energy is characterized as the energy difference between the reactants and the final product, while the activation barrier represents the energy gap that must be overcome to reach the transition state (TS) from the initial reactants. So, the desorption barrier could be estimated by the difference between the activation barrier and reaction energy.

It is known that the change in Gibbs free energy (ΔG) can quantitatively delineate the reactivity of hydrogen molecule dissociation on Ti-doped B_7_ clusters. It can be calculated using the following equation:

(1) $$\Delta G=\Delta E+\Delta E_{\mathrm{zp}}-T\Delta S,$$

where the variables *E*, $E_{\mathrm{zp}}$, *T*, and *S* represent the total energy, zero-point vibrational energy, temperature (set at 298.15 K), and entropy, respectively. In Equation (1), ΔG is used to calculate the reaction energy between the products and reactants, as well as the activation barrier between the TS and the reactants. The calculated reaction energies and activation barriers are also presented in Figure 3. Each TS exhibits a significant imaginary frequency, indicating that it is located at a saddle point on the potential energy surface, as demonstrated in the Supporting Information.

We found that the H_2_ molecule dissociation on TiB_7_ cluster is exothermic with negative reaction energies of –38.05 to –12.10 kcal/mol and activation barriers of 12.86 to 24.79 kcal/mol. Compared to other clusters, such as TiMg_5_ with an activation barrier of 9.52 kcal/mol [33], the activation energy barrier for the TiB_7_ cluster is not low. This indicates that, although hydrogen molecules may dissociate on the TiB_7_ cluster to form the following TiB_7_(H)_6_ and TiB_7_(H)_7_ clusters, this dissociation process is not particularly facile. The reaction pathway for dissociated H atoms adsorbing onto Ti atoma exhibits the highest activation barrier, followed by those adsorbing onto B1 atoms, and the lowest barriers are observed when adsorbing onto B2 atoma. This indicates that the activation barriers are not only related to the type of the adsorbed atom, but also to the chemical environment in which the adsorbed atom is located (such as charge, as detailed in Table 1). We also note that the desorption of H atoms into free H_2_ molecules necessitates overcoming a high desorption barrier ranging from 36.88 to 53.12 kcal/mol.

**Figure 4:**
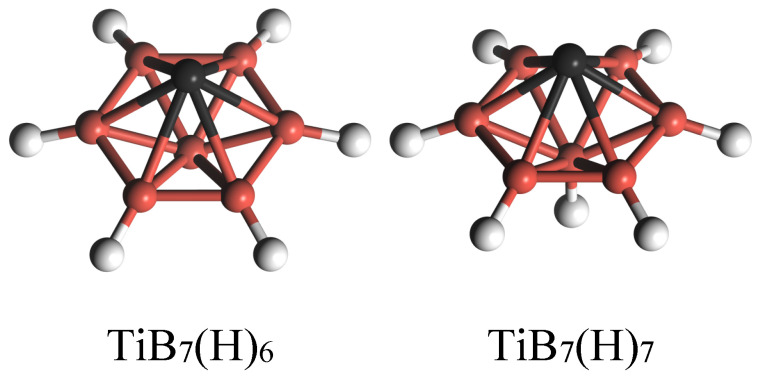
Equilibrium structures of TiB_7_(H)_6_ and TiB_7_(H)_7_ clusters.

In view of the fact that H_2_ molecules can dissociate on TiB_7_ clusters to form hydride clusters, two hydride clusters, TiB_7_(H)_6_ (six H atoms doped on B1 atoms) and TiB_7_(H)_7_ (six H atoms doped on B1 atoms and one H atom doped on B2 atom), as shown in Figure 4, as well as a TiB_7_ cluster, were considered in our subsequent research on the adsorption of H_2_ molecules. Hydrogenation can alter the electronic properties of host TiB_7_ clusters and may improve their hydrogen storage capabilities [34].

The Hirshfeld charge population was calculated to understand and interpret the charge transfer process. Table 1 summarizes the atomic charges of reactants, transition states, and products of the four reaction processes. From the table, it is evident that for all structures, the Ti atom loses charge, with an amount ranging from approximately 0.491e to 0.599e, while the B1 atom gains charge. This aligns with the conclusion in the literature that charge transfer occurs from transition metal atoms to boron atoms [35]. The B2 atom, situated between B1 atoms and farther away from the Ti atom, does not receive charge from the Ti atom but instead transfers a small amount of charge to B1. In the case of H atoms in the reactants, due to their relatively large distance from the TiB_7_ cluster, they are generally uncharged or carry a small amount of charge. However, H atoms in the products acquire a small number of electrons. For transition states, except in Pathway 4, H atoms lose a minor amount of charge. Charge transfer is an inevitable consequence of orbital hybridization between atoms, which not only facilitates the formation of stable clusters but also promotes subsequent hydrogen molecule adsorption [36].

#### 2.2.2. Adsorption of H_2_ Molecule on TiB_7_ Cluster

**Figure 5:**
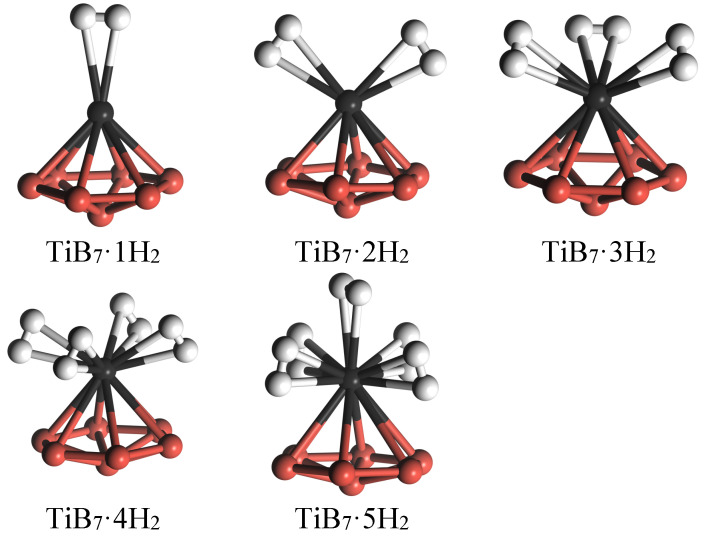
The equilibrium structures for H_2_ molecular adsorption on the TiB_7_ cluster.

Regarding the hydrogen adsorption on the TiB_7_ cluster, we systematically introduced one to six H_2_ molecules onto the Ti atom. The subsequent geometric optimizations revealed that the Ti atom can accommodate up to five H_2_ molecules in molecular form, while the redundant H_2_ molecule is rejected. Figure 5 shows the equilibrium structures for H_2_ molecular adsorption on the TiB_7_(H)_6_ cluster, indicating that the TiB_7_ cluster adsorbs hydrogen in the molecular form. Consequently, the TiB_7_ cluster has the capacity to store up to five H_2_ molecules, yielding a peak hydrogen storage mass fraction of 7.5%. The hydrogen storage mass fraction is slightly lower than that of the ScB_*n*_ (*n* = 1–12) clusters (9.11 wt%) [26], but higher than those of Y-doped B_40_ (5.8 wt%) [37] and Ti-decorated B_38_ fullerene (7.44 wt%) [23]. Furthermore, it exceeds the target (5.5 wt%) set by the US Department of Energy for the year 2020.

The average bond lengths *d* of H–H, Ti–H, and Ti–B of ${\mathrm{TiB}}_{7}\cdot$ *n*H_2_ (*n* = 1–5) clusters are listed in Table 2. Around the positive charged Ti atom, H_2_ molecules are polarized and attached to the TiB_7_ cluster, and their interatomic distances are elongated by a range of 0.02 to 0.05 Å compared to the 0.75 Å found in an isolated H_2_ molecule. An examination of the data reveals that the average length of H–H bonds shows an increasing trend, while that of Ti–H bonds exhibits a decreasing trend. These observations suggest that as the amount of adsorbed H_2_ molecules increases, the interaction between the Ti atom and H_2_ molecules becomes stronger, thereby intensifying the degree of activation within the H_2_ molecules.

The capability of clusters to adsorb and release H_2_ molecules can be evaluated through their binding energy. The average binding energy ($E_{\mathrm{ad}}$) associated with H_2_ molecules is determined by

(2) $$E_{\mathrm{ad}}=\left[E\left({\mathrm{TiB}}_{7}{\left(H\right)}_{m}\right)+nE\left(H_{2}\right)-E\left({\mathrm{TiB}}_{7}{\left(H\right)}_{m}\cdot nH_{2}\right)\right]/n,$$

where *E*(TiB_7_(H)_*m*_), *E*(H_2_), and *E*(TiB_7_(H)_*m*_·*n*H_2_) are the total energy of TiB_7_(H)_*m*_, H_2_, and TiB_7_(H)_*m*_·*n*H_2_, respectively. Typically, zero-point vibration energy is omitted in calculations of binding energy [38,39,40]. So, the $E_{\mathrm{ad}}$ here does not include the zero-point vibration energy.

The computed average binding energies for H_2_ molecules in the TiB_7_·*n*H_2_ (*n* = 1–5) series fell within the range of 0.32 to 0.27 eV. These values are indicative of a state that lies between physical adsorption and chemical adsorption, making them optimal for the reversible storage and release of hydrogen at conditions close to ambient temperatures and moderate pressures [7].

#### 2.2.3. Adsorption of H_2_ Molecule on TiB_7_(H)_6_ and TiB_7_(H)_7_ Clusters

Based on the above analysis, H_2_ molecules can dissociate on the B atom of the TiB_7_ cluster to form a hydride cluster. To hinder the dissociation of H_2_ moleculse on the TiB_7_ cluster, we passivated the TiB_7_ cluster by introducing B–H bonds, which may benefit retrievable hydrogen storage and fast kinetics [6]. For this purpose, TiB_7_(H)_6_ and TiB_7_(H)_7_ clusters were readily constructed via the introduction of six or seven H atoms, as shown in Figure 4. One can find that the equilibrium Ti–B2 bond distances in TiB_7_(H)_6_ and TiB_7_(H)_7_ were 2.28 Å and 2.30 Å, respectively, which are comparable to that in the TiB_7_ cluster (2.29 Å), indicating that the effect of passivated hydrogen on the geometric structure of host cluster was insignificant.

**Figure 6:**
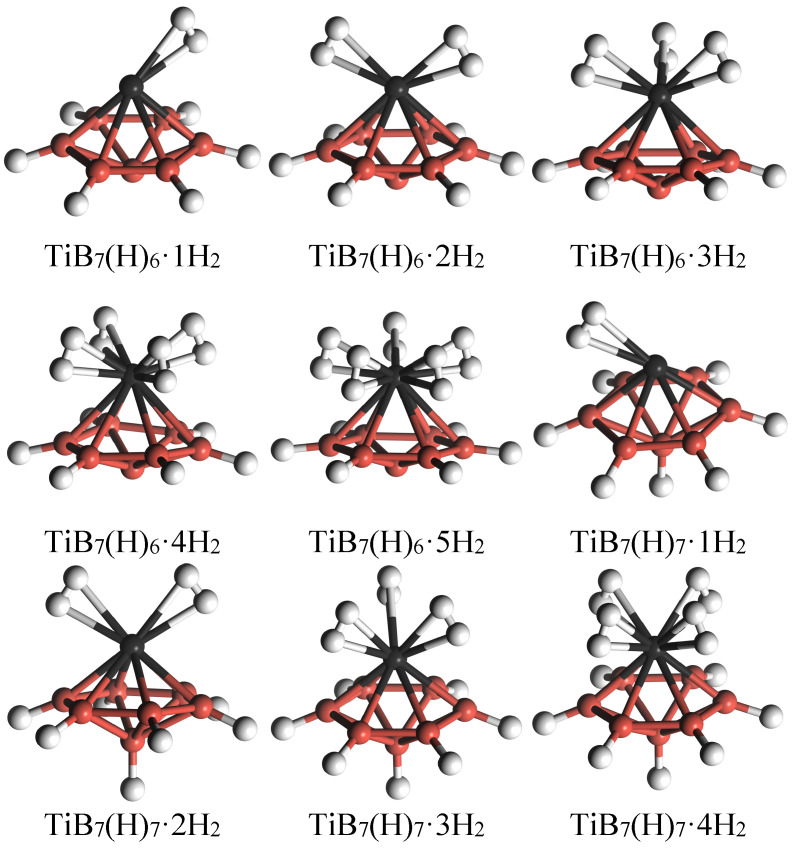
Equilibrium structures for H_2_ molecular adsorption on TiB_7_(H)_6_ and TiB_7_(H)_7_ clusters.

In Figure 6, we present all of the equilibrium structures for H_2_ molecular adsorption on the TiB_7_(H)_6_ and TiB_7_(H)_7_ clusters. Similar to the TiB_7_ cluster, the TiB_7_(H)_6_ cluster can store one to five H_2_ molecules around the Ti atom, yielding a maximum hydrogen storage mass fraction of 7.2%. It is noteworthy that the H–H bond in the TiB_7_(H)_6_·*n*H_2_ (*n* = 1–5) cluster was elongated to a range of 0.79–0.83 Å, which is larger than that in the TiB_7_·*n*H_2_ (*n* = 1–5) cluster. Despite this, the adsorbed hydrogen in TiB_7_(H)6·*n*H_2_ (*n* = 1–5) remained in the molecular form. With further H_2_ molecule adsorption, $E_{\mathrm{ad}}$ of TiB_7_(H)6·*n*H_2_ (*n* = 1–5) varies between 0.44 eV and 0.50 eV, comfortably situated within the optimal range of 0.2–0.6 eV for reversible hydrogen storage applications [7].

Finally, we investigate the interaction between H_2_ molecules and the TiB_7_ cluster. The findings indicate that the TiB_7_(H)_7_ cluster can accommodate a maximum of four H_2_ molecules with an adsorption energy ($E_{\mathrm{ad}}$) ranging from 0.35 to 0.40 eV, corresponding to a peak hydrogen storage mass fraction of 5.8%. In summary, the incorporation of passivated hydrogen atoms altered the electronic properties of the TiB_7_ cluster, thereby strengthening the interaction between the adsorbed hydrogen molecules and the TiB_7_ cluster.

It is widely acknowledged that boron nanomaterials, encompassing small clusters, nanotubes, and sheets exhibit a propensity for adopting planar or quasi-planar configurations. These configurations are predominantly constructed from hexagonal or pentagonal pyramid units [10,41,42,43]. Within the TiB_7_ cluster, the quasi-planar B_7_ unit can be perceived as fragments derived from boron fullerenes, nanotubes, and sheets. Hence, the findings of this study are anticipated to inform the development of other boron nanomaterials, serving as a guide for researchers in designing and synthesizing these materials to ensure their properties are optimized for practical application.

### 2.3. Electronic Properties

We now delve into the H_2_ molecular adsorption mechanism in the TiB_7_ cluster. The Hirshfeld charge population was calculated to investigate the charge transfer between Ti and B atoms. The results show that the electrons transfer from the Ti atom (0.59 e) to B1 atoms (–0.10 e/atom), generating an induced electric field in the region between the Ti atom and boron atoms. This electric field will polarize the covalent bonds of surrounding H_2_ molecules [44], leading to a weakly electrostatic interaction between these H_2_ molecules and the TiB_7_ cluster. For the TiB_7_(H)_6_ and TiB_7_(H)_7_ clusters, we can draw similar conclusions. Consequently, the positively charged exposed Ti atom likely serves as the reaction center, enhancing the adsorption capacity of H_2_ molecules.

**Figure 7:**
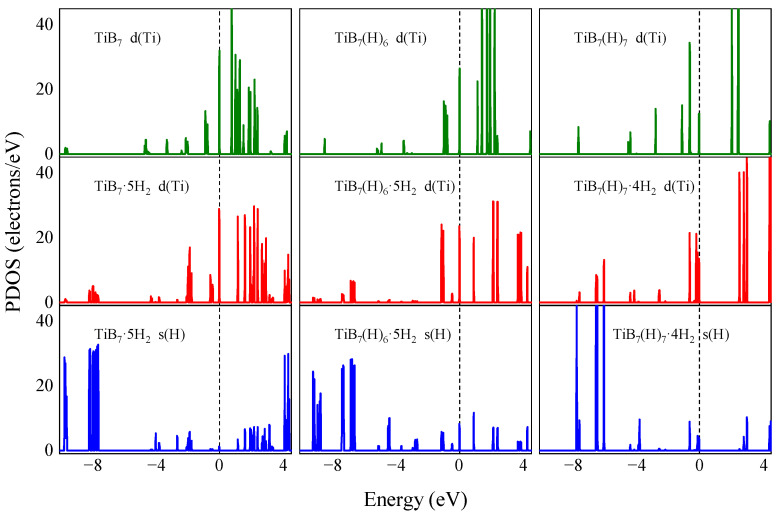
The partial densities of states (PDOS) of TiB_7_, TiB_7_·5H_2_, TiB_7_(H)_6_, TiB_7_(H)_6_·5H_2_, TiB_7_(H)_7_, and TiB_7_(H)_7_·4H_2_ clusters. The vertical dashed line is Fermi level.

To gain a deeper understanding of the bonding characteristics between the H_2_ molecules and the TiB_7_ or TiB_7_(H)_6_ or TiB_7_(H)_7_ cluster, as a particular case, Figure 7 displays the partial densities of states (PDOS) for the Ti *d* and H_2_ molecular *s* orbitals in the TiB_7_·5H_2_ cluster, as well as those for the Ti *d* orbital in the TiB_7_ cluster. The Fermi energy ($E_{f}$) is set to zero and is depicted by the vertical dashed line in the figure. In the TiB_7_·5H_2_ cluster, the peaks observed around −8 eV for the H_2_ molecule’s *s* orbitals correspond to the σ bonding states, whereas the peaks situated around 2 eV represent the σ* antibonding states. The former is dominant, which again demonstrates the H–H bonds are not broken. In comparison to the TiB_7_ cluster, new peaks of Ti *d* orbitals in the TiB_7_·5H_2_ cluster appear around −8 eV, which correspond to the hybridization of the Ti *d* with the σ orbitals of H_2_ molecules. This hybridization interaction mechanism is called the Kubas interaction [45,46]. According to molecular orbital theory, there are both bonding and antibonding states in hydrogen molecules. When the H_2_ molecule is closed to the TiB_7_ cluster, it donates σ electrons into vacant *d* orbitals of the Ti atom. At the same time, the Ti atom donates occupied *d* electrons back into the empty σ* antibonding orbitals, leading to a strong interaction between the H_2_ molecules and the TiB_7_ cluster [32,47]. Figure 7 also shows the PDOS of TiB_7_(H)_6_ and TiB_7_(H)_7_ before and after H_2_ adsorption, wherein we can observe similar results.

In summary, both polarization and hybridization play crucial roles in the adsorption of H_2_ molecules on the TiB_7_ cluster, as extensively reported by previous research works [48].

## 3. Computational Details

All computations were performed within the framework of the generalized gradient approximation (GGA) in DFT, utilizing the DMOL_3_ code [49]. To determine an appropriate functional for describing the exchange and correlation effects of electrons, we initially calculated spin multiplicity, vibrational frequencies, and bond lengths of the dimer B_2_ cluster using various functionals. As presented in Table 3, the Perdew–Burke–Enzerhof (PBE) functional [50], augmented with a revised version of long-range Grimme dispersion correction [51], was ultimately chosen for all calculations due to its close alignment with experimental data [52].

The electronic configuration was determined through the self-consistent solution of spin-unrestricted Kohn–Sham equations, adhering to a stringent convergence criterion of $10^{-6}$ Hartree for the total energy. Using double numerical polarized (DNP) basis sets, an orbital cutoff radius of 5.5 Å was carefully maintained. The parameters for geometric optimization convergence were set at $10^{-5}$ Hartree for total energy, 0.02 Hartree/Å for force, and 0.05 Å for displacement. To accelerate the self-consistent field (SCF) convergence, thermal smearing at 0.002 Hartree was implemented. Additionally, frequency analyses were conducted to verify that all structures derived from our computations represented local minima on the potential energy surface.

During our investigation into the mechanisms of adsorption and dissociation, we employed the nudged elastic band (NEB) methodology. This approach was grounded in the findings from linear synchronous transit (LST) and quadratic synchronous transit (QST) analyses, as documented in reference [55], to compute the minimum energy path.

## 4. Conclusions

In this study, we conducted a thorough investigation of the Ti-doped B_7_ cluster, focusing on its geometry, stability, electronic properties, and hydrogen storage capabilities through extensive first-principles calculations. Our results demonstrate the thermodynamic stability of the ground-state TiB_7_ cluster at temperatures above room temperature (300 K and 400 K), as evidenced by ab initio molecular dynamic simulations.

Regarding hydrogen storage, we found that the TiB_7_ cluster exhibits remarkable potential. Specifically, the Ti atom within the cluster can adsorb up to five H_2_ molecules, achieving a maximum hydrogen storage mass fraction of 7.5%. Notably, even when the cluster is hydrated to form TiB_7_(H)_6_ and TiB_7_(H)_7_, it can still adsorb up to five and four H_2_ molecules, respectively, yielding hydrogen storage mass fractions of 7.2% and 5.8%. These findings are supported by the calculated average adsorption energies of H_2_ molecules, which lie within the optimal range of 0.27 to 0.50 eV, indicating that these structures are well suited for reversible hydrogen storage under moderate temperature and pressure conditions.

Furthermore, our analysis of PDOS reveals that the adsorption of H_2_ molecules onto the TiB_7_ cluster is facilitated by the hybridization between the empty Ti *d* orbitals and the σ orbitals of the H_2_ molecule, combined with polarization effects. This mechanism provides a deeper understanding of the hydrogen storage capabilities of the TiB_7_ cluster.

In summary, our research suggests that the TiB_7_ cluster holds significant promise as a hydrogen storage material under conditions close to ambient. While further experimental investigations are needed to validate these findings, our results provide an important step forward in understanding the adsorption and dissociation of hydrogen molecules on TiB_7_ clusters, paving the way for potential practical applications in hydrogen storage.

## Figures and Tables

**Table 1:** The atomic charges of reactants, transition states, and products of the four reaction processes. The charges of B1 and H atoms are averaged values.

	Pathway 1			Pathway 2			Pathway 3			Pathway 4		
	**Reactant**	**TS**	**Product**	**Reactant**	**TS**	**Product**	**Reactant**	**TS**	**Product**	**Reactant**	**TS**	**Product**
B1	−0.101	−0.105	−0.080	−0.096	−0.112	−0.095	−0.097	−0.113	−0.081	−0.099	−0.050	−0.056
B2	0.017	−0.036	−0.019	0.015	−0.007	0.042	0.015	0.000	0.011	0.010	0.019	0.034
Ti	0.587	0.518	0.561	0.591	0.519	0.599	0.591	0.552	0.558	0.491	0.494	0.524
H	0.000	0.074	−0.031	−0.014	0.079	−0.035	−0.011	0.064	−0.041	0.048	−0.106	−0.111

**Table 2:** Average bond lengths (*d*, Å) of H–H, Ti–H, and Ti–B of ${\mathrm{TiB}}_{7}\cdot$*n*H_2_ (*n* = 1–5) clusters.

	${\mathrm{TiB}}_{7}\cdot$1H_2_	${\mathrm{TiB}}_{7}\cdot$2H_2_	${\mathrm{TiB}}_{7}\cdot$3H_2_	${\mathrm{TiB}}_{7}\cdot$4H_2_	${\mathrm{TiB}}_{7}\cdot$5H_2_
*d*_H–H_	0.767	0.781	0.789	0.785	0.800
*d*_Ti–H_	2.221	2.121	2.049	2.080	2.015
*d*_Ti–B_	2.306	2.321	2.420	2.400	2.517

**Table 3:** Comparisons between calculational spin multiplicity (*SM*), frequency (ν, ${\mathrm{cm}}^{-1}$), and bond length (*d*, Å) of B_2_ and Ti_2_ dimers by using different functionals with different dispersion correction (Dc) (if available) and experimental data.

Functional	Dc	${\mathrm{SM}}_{B2}$	$\nu_{B2}$	$d_{B2}$	${\mathrm{SM}}_{\mathrm{Ti}2}$	$\nu_{\mathrm{Ti}2}$	$d_{\mathrm{Ti}2}$
PBE	Grimme	3	1043.0	1.592	3	433.3	1.959
	Tkatchenko–Scheffler	3	1043.1	1.592	3	433.3	1.959
BLYP	Grimme	3	1008.7	1.606	3	436.5	1.971
	Tkatchenko–Scheffler	3	1007.1	1.606	3	432.2	1.975
PW91	Ortmann–Bechstedt–Schmidt	5	1281.0	1.536	3	437.6	1.952
BOP	-	3	990.4	1.612	3	424.2	1.976
BP	-	5	1281.3	1.539	3	429.6	1.956
RPBE	-	3	993.6	1.600	1	421.3	1.983
HCTH	-	3	1251.9	1.598	3	447.5	1.938
PBEsol	-	5	1298.0	1.539	3	455.0	1.925
VWN-BP	-	3	1053.4	1.592	3	429.6	1.955
Experiment	-	-	1051.3 ^a^	1.59 ^a^	3 ^b^	407.9 ^c^	1.9422 ^b^

^a^ Reference [52], ^b^ Reference [53], ^c^ Reference [54].

## Data Availability

Data are contained within the article and Supplementary Materials.

## Supplementary Materials

The following supporting information can be downloaded at: https://www.mdpi.com/article/10.3390/molecules29235795/s1, Table S1: The frequency (ν, ${\mathrm{cm}}^{-1}$) of all structures.

## Abbreviations

The following abbreviations are used in this manuscript:

$E_{f}$	Fermi energy
$E_{\mathrm{ad}}$	Average binding energy
DFT	Density functional theory
GGA	Generalized gradient approximation
PBE	Perdew–Burke–Enzerhof functional
Dc	Dispersion correction
DNP	Double numerical polarized basis
SCF	Self-consistent field
NEB	Nudged elastic band method
LST	Linear synchronous transit
QST	Quadratic synchronous transit
ZPE	Zero-point vibration energy
AIMD	ab initio molecular dynamic simulation
*S*	Entropy
*T*	Temperature
*E*	Total energy
*SM*	Spin multiplicity
ν	Frequency
*NVT*	Constant volume and temperature ensemble
TS	Transition state
PDOS	Partial densities of states
