# Supplementary material for: First-Principles Study of Titanium-Doped B7 Cluster for High Capacity Hydrogen Storage
Source: Molecules. 2024 Dec 7;29(23):5795. doi: 10.3390/molecules29235795 (PMC11643889; doi:10.3390/molecules29235795)
Supplement: Supplementary file 1 [file molecules-29-05795-s001.zip › molecules-3333253-supplementary.pdf]

Table S1: The frequency (ν, cm^{-1}) of all structures.

	ν_1	ν_2	ν_3	ν_4	ν_5
TiB7_6H	294.4	320.3	324.0	333.7	363.1
TiB7_6H_1H2	100.3	283.7	314.0	315.4	332.9
TiB7_6H_2H2	107.9	138.5	246.1	289.4	299.4
TiB7_6H_3H2	115.4	123.0	144.5	249.5	253.7
TiB7_6H_5H2	59.6	173.8	221.7	248.5	296.8
TiB7_7H	226.7	292.6	332.5	346.5	369.9
TiB7_7H_1H2	61.2	197.0	256.4	313.4	331.7
TiB7_7H_2H2	26.1	75.2	169.5	265.4	285.5
TiB7_7H_3H2	45.3	167.4	168.9	252.8	254.5
TiB7_7H_4H2	24.5	214.1	226.9	263.6	279.5
TiB7	172.6	212.9	339.9	415.9	456.9
TiB7_1H2	84.0	142.5	148.1	234.9	256.8
TiB7_2H2	80.8	164.6	189.9	206.4	276.7
TiB7_3H2	76.8	155.0	158.1	219.6	270.7
TiB7_4H2	39.6	164.5	236.1	244.9	252.4
TiB7_5H2	15.9	183.0	206.2	232.3	243.7
Pathway1_Reactant	36.5	52.6	68.2	79.3	137.2
Pathway1_TS	-1046.1	115.3	244.5	385.6	418.1
Pathway1_Product	257.1	301.6	326.8	382.9	389.3
Pathway2_Reactant	4.9	42.2	150.3	209.4	217.1
Pathway2_TS	-829.3	205.2	270.9	304.2	394.8
Pathway2_Product	101.2	212.1	278.6	364.0	373.8
Pathway3_Reactant	16.2	24.0	143.1	160.4	206.1
Pathway3_TS	-801.2	186.1	294.0	301.3	349.3
Pathway3_Product	171.7	263.2	337.1	357.7	408.3
Pathway4_Reactant	66.5	74.4	95.9	202.7	318.5
Pathway4_TS	-629.7	215.0	242.0	309.2	325.5
Pathway4_Product	190.6	263.2	303.0	340.2	388.7
